# Low-Cost and High-Productivity Three-Dimensional Nanocapacitors Based on Stand-Up ZnO Nanowires for Energy Storage

**DOI:** 10.1186/s11671-016-1429-2

**Published:** 2016-04-21

**Authors:** Lei Wei, Qi-Xuan Liu, Bao Zhu, Wen-Jun Liu, Shi-Jin Ding, Hong-Liang Lu, Anquan Jiang, David Wei Zhang

**Affiliations:** State Key Laboratory of ASIC and System, School of Microelectronics, Fudan University, Shanghai, 200433 People’s Republic of China

**Keywords:** Three dimensional, Nanocapacitors, ZnO nanowires, Capacitance density, Energy storage

## Abstract

Highly powered electrostatic capacitors based on nanostructures with a high aspect ratio are becoming critical for advanced energy storage technology because of their high burst power and energy storage capability. We report the fabrication process and the electrical characteristics of high capacitance density capacitors with three-dimensional solid-state nanocapacitors based on a ZnO nanowire template. Stand-up ZnO nanowires are grown face down on p-type Si substrates coated with a ZnO seed layer using a hydrothermal method. Stacks of AlZnO/Al_2_O_3_/AlZnO are then deposited sequentially on the ZnO nanowires using atomic layer deposition. The fabricated capacitor has a high capacitance density up to 92 fF/μm^2^ at 1 kHz (around ten times that of the planar capacitor without nanowires) and an extremely low leakage current density of 3.4 × 10^−8^ A/cm^2^ at 2 V for a 5-nm Al_2_O_3_ dielectric. Additionally, the charge-discharge characteristics of the capacitor were investigated, indicating that the resistance-capacitance time constants were 550 ns for both the charging and discharging processes and the time constant was not dependent on the voltage. This reflects good power characteristics of the fabricated capacitors. Therefore, the current work provides an exciting strategy to fabricate low-cost and easily processable, high capacitance density capacitors for energy storage.

## Background

Even after decades of research, energy storage technology continues to be a major challenge for advanced modern society. The development of small size, lightweight, and environmentally friendly energy storage devices has attracted great attention owing to fast-growing energy demands for portable and wearable electronics [1]. Therefore, energy storage systems including fuel cells, batteries, and supercapacitors are being adapted and optimized with nanostructured components [2]. Among various nanostructure-based energy storage devices, nanocapacitor arrays have been extensively studied for the next generation of energy storage systems because of their moderate energy density and high power density [3].

According to the well-known equation for a parallel-plate capacitor, *C = ε*_0_*ε*_r_*A*/*d*, where *C* is the capacitance, *ε*_0_ and *ε*_r_ are the dielectric constants of the vacuum and dielectric, respectively, *A* is the surface area of the electrode, and *d* is the thickness of the dielectric; the capacitance density can be increased by including high-permittivity dielectrics such as Al_2_O_3_, HfO_2_, and TiO_2_, including various hybrid dielectric stacks to increase *ε*_r_ and using three-dimensional (3D) capacitor designs to achieve a larger capacitor electrode area to increase *A* [4]. Decreasing *d* is not usually an option for capacitors in power applications because this could lead to a higher leakage current and a lower breakdown voltage. Therefore, 3D nanocapacitor arrays with a high aspect ratio are considered to be a robust candidate to achieve a high capacitance density. To date, many methods have been proposed for the fabrication of 3D nanocapacitors, mainly focusing on different nanostructured templates such as anodic aluminum oxide (AAO) [5–11], carbon nanotubes (CNTs) [12, 13], silicon-based nanowires, nanoholes and nanopillars [14–18], and InAs nanowires [19]. The AAO template has been widely used because nanopore arrays exhibit a high degree of regularity and uniformity. However, conventional AAO fabrication needs a relatively long time, the use of resources is inefficient, and the chemicals can be toxic, which restrict their practical application [20]. Although a CNT template has a good electrical conductivity and can be used as capacitor electrodes, the production of CNTs usually needs quite a high growth temperature (>750 °C) [13]. The leakage current characteristics of the CNT-based nanocapacitors are usually unsatisfactory, which is attributed to the top connection of stand-up CNTs when they become long enough, thus resulting in an increase in leakage paths [12]. Silicon-based nanostructures are easily integrated with current silicon technologies. Nevertheless, silicon nanoholes with a high aspect ratio are difficult to etch and the definition of multilayered structure capacitors requires costly lithography processes [16]. The growth of silicon nanowires usually requires a relatively high temperature (>400 °C), which is not suitable for flexible devices [14].

During the past decade, ZnO nanostructures such as nanoparticles, nanorods, nanoforests, and nanowires (NWs) have been investigated intensively for various applications because of their wide bandgap, excellent thermal and chemical stability, and special electrical and optoelectronic characteristics [21–23]. For instance, ZnO nanoforests have been explored for photoelectrochemical applications because of their large surface area [24], and long stand-up ZnO nanowires or nanorods are necessary for piezoelectric devices or solar cells [25, 26]. Nevertheless, there are no reports of using stand-up ZnO NW templates for the fabrication of 3D nanocapacitors. Various synthesis methods of ZnO NWs have been reported such as the vapor-liquid-solid process [27], chemical vapor deposition (CVD) [28], pulse laser deposition [29], and hydrothermal synthesis [30, 31]. Among these growth processes, the hydrothermal synthesis is preferred because of its low cost, low growth temperature, and large growth area and because it is environmentally benign. On the other hand, the selection of electrode materials also plays an important role in the preparation of 3D nanocapacitors. Transparent conducting oxides (TCOs), including indium oxide and aluminum-doped zinc oxide (AZO), have many attractive properties such as good thermal stability, relatively low resistivity, and high transmittance [32, 33]. Introducing TCOs as electrodes of 3D nanocapacitors could make it possible for the integration of energy storage devices and optoelectronic devices.

Therefore, this article reports the preparation of stand-up ZnO nanowires on a silicon substrate using a hydrothermal synthesis. Using the ZnO nanowire as a template and the AZO film as an electrode, high-density 3D solid-state capacitors were fabricated and characterized physically and electrically for energy storage applications.

## Methods

**Fig. 1:**
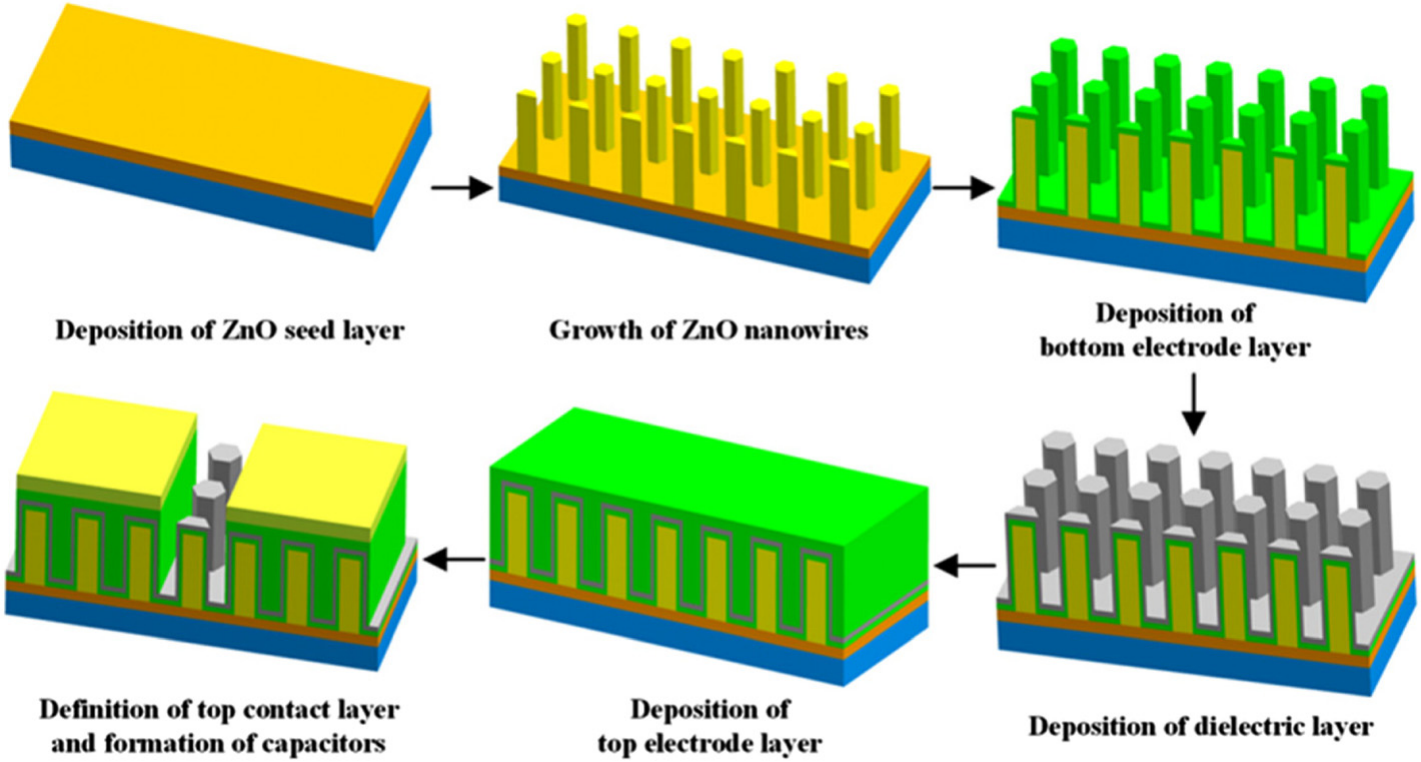
Schematic of the fabrication of the nanocapacitors based on ZnO nanowires

The experimental procedure for the fabrication of the ZnO NW-based nanocapacitor arrays is illustrated schematically in Fig. 1. The whole fabrication procedure included six key steps: deposition of a ZnO seed layer, growth of ZnO nanowires, deposition of a thin bottom electrode layer, deposition of a dielectric layer, deposition of a top electrode layer, definition of a top contact layer, and formation of the capacitors. The detailed process steps are described as follows.

### Growth of the Stand-Up ZnO Nanowires

ZnO nanowires were synthesized via a modified hydrothermal method, which was described in our previous paper [34]. First, p-type Si wafers were cleaned with the standard Radio Corporation of America (RCA) cleaning process, and then, around 18 nm of a ZnO thin film was grown on the Si substrate at 200 °C using atomic layer deposition (ALD). This was then used as the seed layer of ZnO NWs. Zn(C_2_H_5_)_2_ and deionized water (DI water) were employed as the precursors for the ALD ZnO. Subsequently, the ZnO seed layer-coated Si substrate was transferred into a Teflon-lined stainless steel autoclave filled with aqueous solution consisting of 0.025 M zinc nitrate hexahydrate [Zn(NO_3_)_2_·6H_2_O)] and 0.025 M hexamethylenetetramine (C_6_H_12_N_4_). It is worthwhile emphasizing that the ZnO seed layer should be face down in the solution. The autoclave was sealed tightly and kept in a stove at 80 °C for 8 h. After that, the resulting sample was removed from the solution and thoroughly rinsed with DI water. It was then dried using a slow high-purity nitrogen flow.

### Deposition of the Conducting and Insulating Thin Films

First, a 10-nm AZO layer was deposited on the as-grown ZnO NWs using thermal ALD at 200 °C, which served as the bottom electrode layer. Herein, the AZO layer was composed of alternate 20 cycles of ZnO and 1 cycle of Al_2_O_3_, giving a resistivity of 1.8 × 10^−3^ Ω cm. ZnO and Al_2_O_3_ were grown from the Zn(C_2_H_5_)_2_/H_2_O and Al(CH_3_)_3_/H_2_O precursors, respectively. To investigate the effect of dielectric thickness, various thicknesses of Al_2_O_3_ layers (5, 10, 15, and 20 nm) were deposited using ALD at 200 °C, acting as the insulator for the nanocapacitors. After the deposition of Al_2_O_3_, a 150-nm AZO layer was deposited by ALD for the top electrode. Thus, the AZO/Al_2_O_3_/AZO capacitor stack was formed. To ensure full diffusion of the precursor molecules into and the gaseous byproducts out of the gaps among the ZnO NWs, five precursor-pulsing and purging durations were adopted in comparison with those normally used for a flat substrate.

### Formation of the Contact and Definition of Capacitors

A 150-nm Mo layer serving as the top contact was deposited using radio frequency magnetron sputtering, and the shape and size of the contact pad were defined using photolithography and a metal lift-off process. Subsequently, the top AZO film outside the pads was etched using dilute hydrochloric acid, and thus, the separated capacitors consisting of nanocapacitors were formed for electrical characterization.

### Characterization Methods

The top view and cross-sectional morphologies of the as-grown ZnO NWs and those coated with different thin films were characterized with scanning electron microscopy (SEM) (Zeiss SIGMA HD microscope, Germany). The cross-sectional images of the fabricated capacitors were observed using field-emission TEM (FEI Tecnai G2 F20 S-TWIN) with an accelerating voltage of 200 kV. Capacitance-voltage (*C*-*V*) and impedance measurements were carried out on a precision impedance analyzer (4294A; Agilent Technologies, Malaysia). Current-voltage (*I*-*V*) was measured on a semiconductor device analyzer (Agilent B1500A; Agilent Technologies, Japan). Charging-discharging characteristics of the capacitors were measured on a function/arbitrary waveform generator (Agilent 33250A; Agilent Technologies, Germany).

## Results and Discussion

**Fig. 2:**
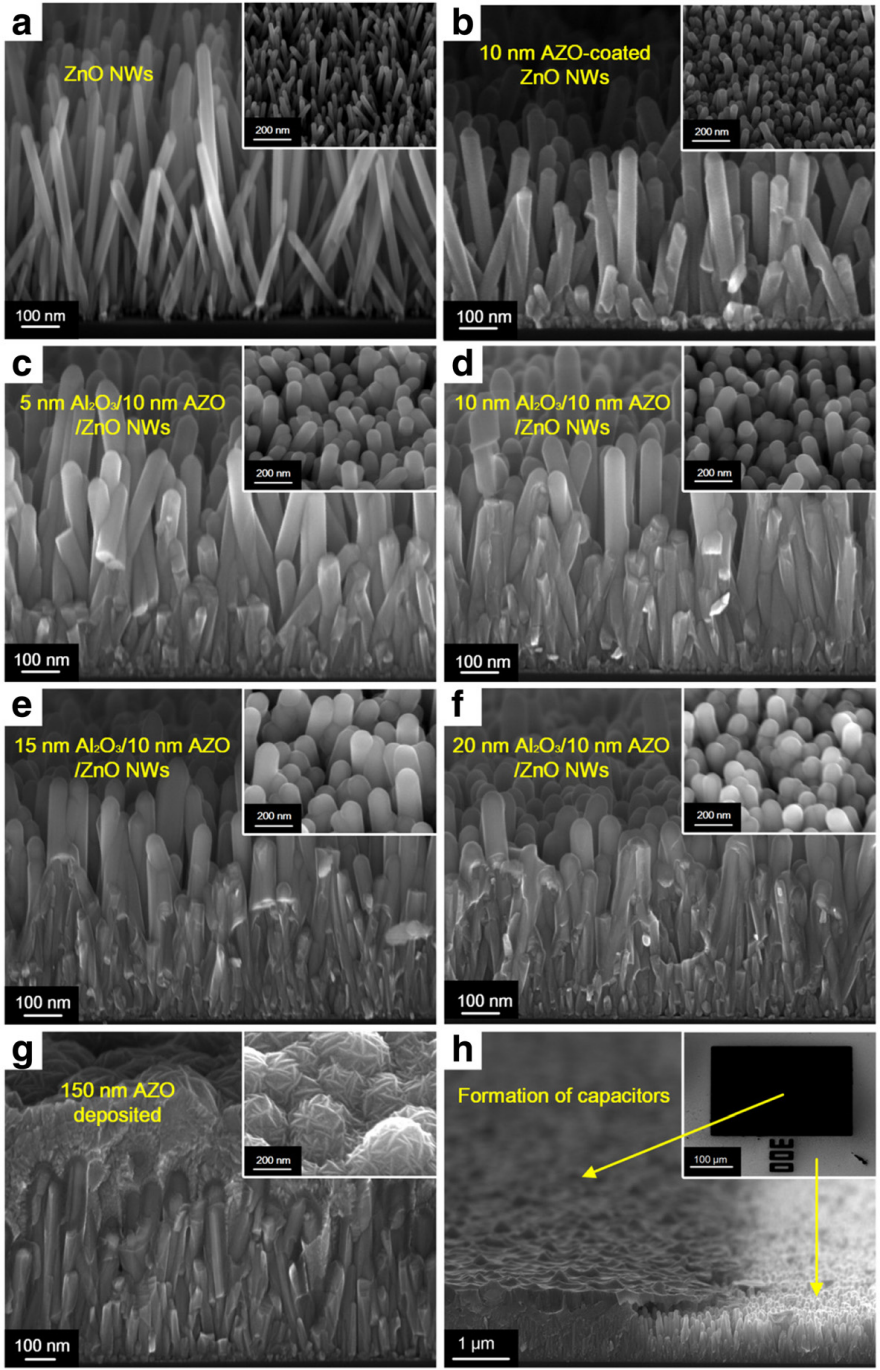
Cross-sectional SEM images of **a** the as-grown ZnO nanowires (NWs), **b** 10-nm AZO-coated ZnO NWs, **c**–**f** ZnO NWs surrounded by 10 nm AZO and 5, 10, 15, and 20 nm Al_2_O_3_, respectively, **g** the structures with deposition of 150 nm AZO acting as the top electrode, **h** the fabricated capacitor with a pad (shown by the *dark* region), and the area without a pad (shown by the *bright* region). The *insert* shows the top view of the corresponding sample at a 50° tilt

Figure 2 shows the cross-sectional and top-view SEM images of the as-grown ZnO NWs and those coated with different thin films formed using ALD as well as the fabricated capacitor. As shown in Fig. 2a, the as-grown ZnO NWs on the seed layer of ZnO stand approximately in an upwards direction with a diameter of 20–30 nm and a height of 500–600 nm. The extracted area density of ZnO NWs is close to 7 × 10^9^ cm^−2^, which is in good agreement with the reported values [23]. ZnO NWs with such a high density and high aspect ratio can guarantee a greatly increased electrode area and hence a significant enhancement in capacitance density. The top view of the ZnO NWs is shown in the inset of Fig. 2a, revealing their high density. After deposition of a 10-nm Al-doped ZnO (AZO) film, the ZnO NWs were coated uniformly with the AZO layer, displaying very smooth surfaces and increased diameters of the NWs, as shown in Fig. 2b. Subsequently, different thicknesses of Al_2_O_3_ films were deposited on the NWs, serving as the dielectric of the nanocapacitor, as illustrated in Fig. 2c–f. As the thickness of Al_2_O_3_ increased from 5 to 20 nm, the gaps in the NWs were increasingly full. The diameters of the coated NWs showed an increasing trend, clearly demonstrated in the inserts of Fig. 2c–f. After the deposition of a 150 nm AZO layer acting as the top electrode, the NWs were completely covered, as shown in Fig. 2g. The top-view SEM image reveals a rough top surface likely caused by the different heights of the ZnO NWs (see the insert of Fig. 2g). Finally, the fabricated capacitor with a Mo contact pad is shown in Fig. 2h, which actually consists of many nanocapacitors. Although irregular margins of the pad/AZO electrode can be observed in Fig. 2h, which could result from a lateral etching effect and/or the lift-off process, they can be ignored compared with the large area of the electrode.

**Fig. 3:**
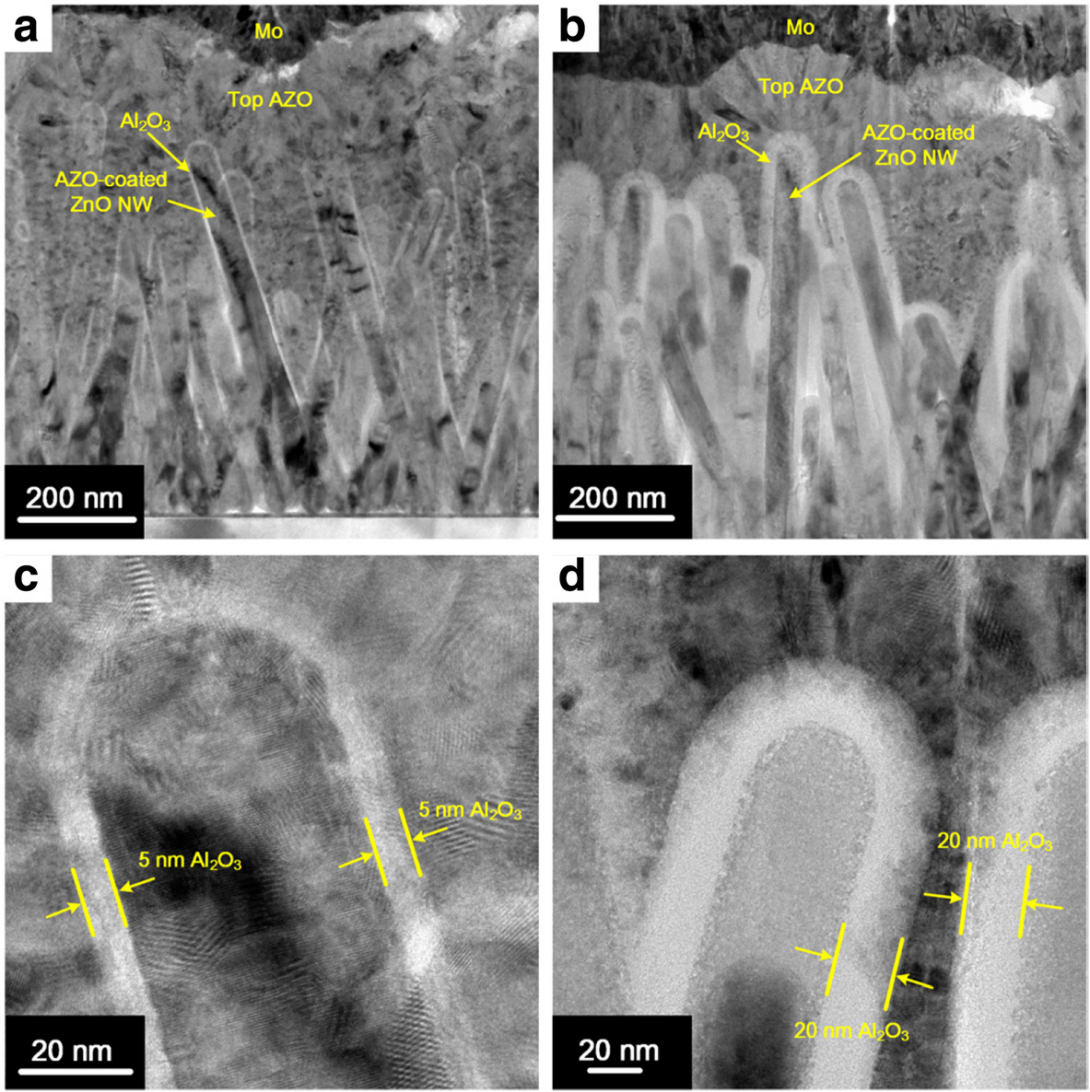
Cross-sectional TEM images of the fabricated nanocapacitors with **a** 5 nm and **b** 20 nm Al_2_O_3_ dielectric layers, respectively. Magnified cross-sectional TEM images of the nanocapacitors with **c** 5 nm of Al_2_O_3_ and **d** 20 nm of Al_2_O_3_

Figure 3 shows the cross-sectional transmission electron microscopy (TEM) images of the fabricated capacitors with 5 and 20 nm of Al_2_O_3_ dielectric layers, respectively. It was found that each capacitor contains many 3D nanocapacitors based on the stand-up ZnO NW template, as shown in Fig. 3a, b. Additionally, the stand-up NWs are coated uniformly and conform to an Al_2_O_3_ dielectric layer, and the thickness of Al_2_O_3_ was accurately controlled, as shown in Fig. 3c, d.

**Fig. 4:**
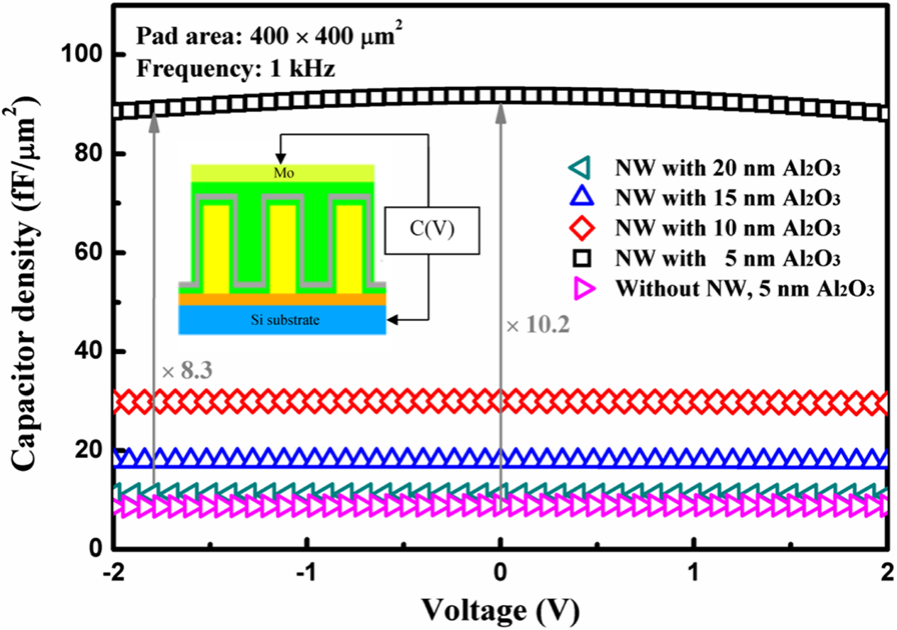
Typical capacitance-voltage (*C*-*V*) characteristics of the ZnO NW-based capacitors with different thicknesses of the Al_2_O_3_ dielectric at 1 kHz. As a comparison, the *C*-*V* curve of the planar capacitor with a 5-nm Al_2_O_3_ dielectric is also illustrated. The *inset* shows the schematic of the electrical test structure

Figure 4 shows the typical capacitance density as a function of voltage at 1 kHz for the ZnO NW-based capacitors with an Al_2_O_3_ dielectric layer ranging from 5 to 20 nm in comparison with the planar capacitor with a 5-nm Al_2_O_3_ dielectric. The C-V curves exhibited a shape typical of metal-insulator-metal capacitors, which was attributed to the metal-like AZO films with resistivity of 1.8 × 10^−3^ Ω cm. The inset shows a schematic of the electrical test structure, which illustrates that the AZO/Al_2_O_3_/AZO multi-layers act as a metal-insulator-metal structure, and the bulk of the ZnO NW/seed layer/Si substrate serves as a series resistor. As the thickness of Al_2_O_3_ increased gradually from 5 to 20 nm, the resulting capacitance density decreased. In theory, the capacitance density should increase four times as the thickness of Al_2_O_3_ decreases from 20 to 5 nm. However, in terms of the 5 nm Al_2_O_3_, a capacitance density as large as 92 fF/μm^2^ was achieved at zero voltage, which is about ten times that of the planar capacitor with 5 nm of Al_2_O_3_, and it is around eight times that (11 fF/μm^2^) of the 20 nm Al_2_O_3_. This deviates seriously from the theoretical value, which can be explained as follows. When the Al_2_O_3_ dielectric layer became very thick, the gaps in the NWs were easily filled; thus, the real contact area between the electrode and the dielectric layer decreased. This reduces the capacitance density. However, the theoretical capacitance of the fabricated capacitor was also estimated by considering each capacitor as an array of parallel cylindrical nanocapacitors. For a single nanocapacitor, the capacitance can be approximated by that of a cylindrical capacitor, described in Eq. (1) [35]:

1 $$ {C}_{\mathrm{single}}=2\uppi {\varepsilon}_0{\varepsilon}_{\mathrm{r}}h/ \ln \left(b/a\right), $$

where ε_0_ is the permittivity in vacuum, *ε*_r_ is the relative dielectric constant of Al_2_O_3_ (*ε*_r_ *=* 8.9), *h* is the average height of the ZnO NWs (*h* = 550 nm), *a* is the average diameter of the ZnO NWs coated with the AZO layer (*a* = 45 nm), and *b* is the coaxial diameter of the Al_2_O_3_ dielectric layer. If the parallel nanocapacitor array is taken into consideration, the total capacitance density is given by Eq. (2):

2 $$ {C}_{\mathrm{d}}=2\uppi {\varepsilon}_0{\varepsilon}_{\mathrm{r}}hd/ \ln \left(b/a\right), $$

where the additional factor, *d*, is the area density of the nanowires. In this case, *d* = 7 × 10^9^ cm^−2^. Regarding the 5-nm Al_2_O_3_ dielectric layer, *b* was calculated to be 55 nm; thus, the estimated capacitance density is about 94.9 fF/μm^2^, which is close to the experimental value of 92 fF/μm^2^.

**Fig. 5:**
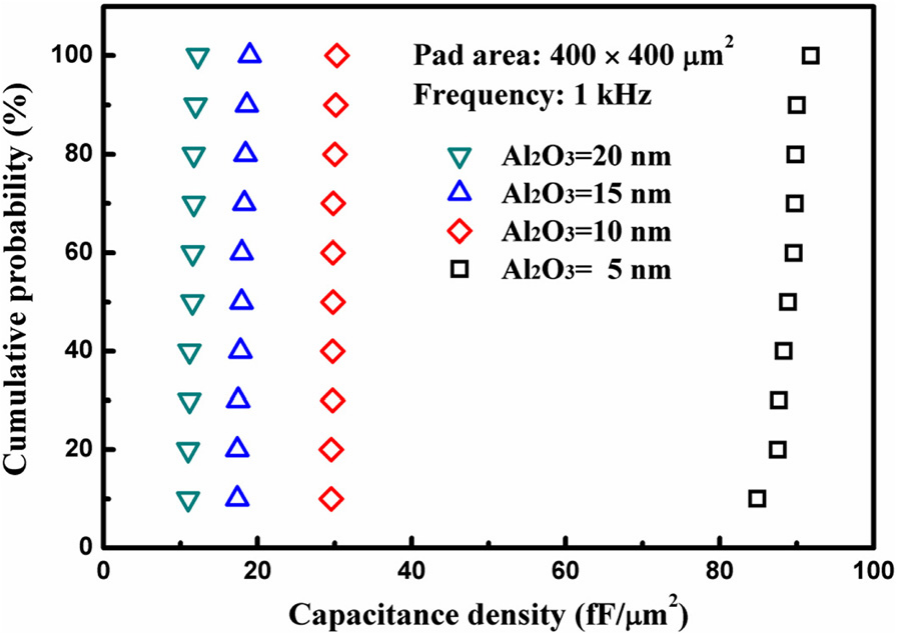
Distribution characteristics of the capacitance densities measured at 0 V and at 1 kHz for the fabricated capacitors with different thicknesses of Al_2_O_3_

To observe the uniformity of the capacitance density of the fabricated capacitors, ten capacitors for each thickness of Al_2_O_3_ were randomly selected for *C*-*V* measurements at 1 kHz. Figure 5 shows the cumulative distribution characteristics of the capacitance densities for capacitors with 5-, 10-, 15-, and 20-nm Al_2_O_3_ layers at 1 kHz and zero voltage. With respect to the 5-nm Al_2_O_3_ layer, the resulting capacitance density has a narrow distribution ranging from 85 to 92 fF/μm^2^; the capacitance density at a 50 % cumulative probability is as large as 89 fF/μm^2^. As the thickness of Al_2_O_3_ increases from 10 to 20 nm, the distribution of the resulting capacitance densities becomes much narrower. This indicates that our fabricated capacitors have quite good electrical uniformity, which relates to the uniform ZnO NWs.

**Fig. 6:**
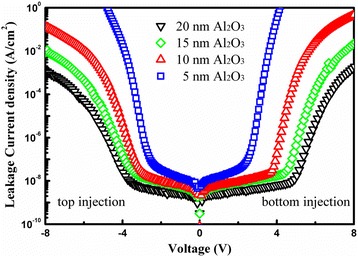
Dependence of the leakage current density on voltage for the fabricated capacitors with different thicknesses of Al_2_O_3_ (5, 10, 15, and 20 nm) at room temperature. All the current-voltage measurements were performed on capacitors with a footprint size of 100 μm × 100 μm

Figure 6 illustrates the leakage current density as a function of voltage for the fabricated capacitors with various thicknesses of Al_2_O_3_ at room temperature. The leakage current density (defined as the leakage current/the footprint area) decreases from 3.4 × 10^−8^ to 3.5 × 10^−9^ A/cm^2^ at 2 V as the thickness of Al_2_O_3_ increases from 5 to 20 nm. If the real contact area between the Al_2_O_3_ dielectric and the AZO electrode (denoted by RA) is taken into account, the resulting leakage current density should be even smaller. For example, for the capacitor with a 5-nm Al_2_O_3_ dielectric layer, using the capacitance obtained from the *C*-*V* measurements and the equation of a parallel plate capacitor, we extracted a real electrode area of 5.7 × 10^4^ μm^2^, which is more than five times the footprint area (100 × 100 μm^2^). Therefore, the real leakage current density was calculated to be 6 × 10^−9^ A/cm^2^, which is remarkably superior to most of the reported results. Such a low leakage current density was attributed to the high-quality Al_2_O_3_ dielectric and superior interfaces of AZO/Al_2_O_3_, which were formed by successive ALD of AZO and Al_2_O_3_ layers without breaking the vacuum.

To compare the current work with other reports on nanocapacitors, Table 1 shows the characteristics of various reported nanocapacitors. Banerjee et al. [5] reported the AAO template-based electrostatic nanocapacitors on a glass substrate, which exhibited a very high equivalent planar capacitance density (EPCD) and a small leakage current density. However, the corresponding capacitance was measured at a frequency as low as 20 Hz. As the authors mentioned, their capacitors showed noticeable capacitance dispersion when the measurement frequency was higher than 100 Hz, thus leading to a decrease in capacitance [5]. Both the transfer and bonding of the AAO template onto other substrates are time-consuming and delicate operations. Klootwijk et al. [16] demonstrated an EPCD of 440 fF/μm^2^ using ALD multiple layers of TiN/Al_2_O_3_ inside silicon trenches. The fabricated capacitor exhibited a relatively high leakage current density. However, the fabrication of this type of nanocapacitor needs multiple lithography and etching steps, especially for the reactive ion etching process for the Si trenches with a high aspect ratio. These definitely increase the fabrication cost. Although Morel et al. [14] reported Si nanowire-based nanocapacitors with an EPCD of 180 fF/μm^2^, the leakage current density was not satisfactory. This is likely because of the diffusion of the copper catalyst incorporated in the growth of the Si nanowires. It also needed a relatively high temperature (425 °C) for the growth of the Si NWs. Briefly, compared with other nanocapacitors based on various templates, our capacitor with 5 nm of Al_2_O_3_ has a high EPCD up to 92 fF/μm^2^ and an extremely low leakage current density of 3.4 × 10^−8^ A/cm^2^ at 2 V. Most importantly, easily processable ZnO NWs were adopted as a template for our nanocapacitors, which have some advantages such as the low cost, easy scale up, and low thermal budget. Furthermore, by increasing the aspect ratio of ZnO NWs and/or using an insulator with a higher dielectric constant, the EPCD can be further enhanced.

**Table 1:** Comparison of the various nanostructured template-based nanocapacitors, including the capacitor structure, material, and fabrication method, capacitance density, and leakage current density. Here, FA and RA represent the extracted leakage current density according to the footprint area and real electrode area, respectively, and the extracted capacitance density means the equivalent planar capacitance density (EPCD). BE and TE are abbreviations of the bottom electrode and top electrode, respectively

Reference	Template	Film deposition	BE	Insulator (thickness)	TE	EPCD (frequency)	Leakage current density
Our work	ZnO NWs	ALD	AZO	Al_2_O_3_ (5 nm)	AZO	92 fF/μm^2^ (1 kHz)	3.4 × 10^−8^ A/cm^2^ at 2 V (FA)
							6 × 10^−9^ A/cm^2^ at 2 V (RA)
Banerjee et al. [5]	AAO	ALD	TiN	Al_2_O_3_ (6.7 nm)	TiN	1 pF/μm^2^ (20 Hz and d.c.)	5.0 × 10^−9^ A/cm^2^ at 2 V (RA)
Klootwijk et al. [14]	Si trench	ALD	TiN	Al_2_O_3_ (10 nm)	TiN	440 fF/μm^2^ (10 kHz)	0.1~1 × 10^−5^ A/cm^2^ at 2 V (FA)
Morel et al. [12]	Si nanowires	CVD/ALD	Si	Al_2_O_3_ (10 nm)	TiN	180 fF/μm^2^ (1 kHz)	1.8 × 10^−7^ A/cm^2^ at 1 V (RA)
							4.0 × 10^−6^ A/cm^2^ at 1 V (FA)
Chang et al. [15]	Si nanopillar	PVD	N^+^-Si	SiO_2_ (5 nm)	Ni	43 fF/μm^2^ (1 kHz)	1.15 × 10^−5^ A/cm^2^ at 2 V (FA)
Zhang et al. [8]	AAO	ALD	AZO	Al_2_O_3_ (10 nm)	AZO	37 fF/μm^2^ (10 kHz)	1.7 × 10^−7^ A/cm^2^ at 1 V (FA)
Kemell et al. [16]	Si trench	ALD	Si	Al_2_O_3_ (50 nm)	AZO	2~25 fF/μm^2^ (10 kHz)	3 × 10^−5^ A/cm^2^ at 2.5 V (RA)
							1.5 × 10^−3^ A/cm^2^ at 2.5 V (FA)
Li et al. [9]	AAO	ALD	AZO	Al_2_O_3_ (10 nm)	AZO	15.3 fF/μm^2^ (100 kHz)	Not satisfactory (no data)
Jang et al. [10]	CNT	PECVD	CNT/Nb	Si_3_N_4_ (65 nm)	Al	6.3 fF/μm^2^ (not given)	2 × 10^−6^ A/cm^2^ at 1 V (RA)
Sohn et al. [6]	AAO	CVD	Al	Al_2_O_3_ (27 nm)	CNT	1.74 fF/μm^2^ (100 kHz)	No data

**Fig. 7:**
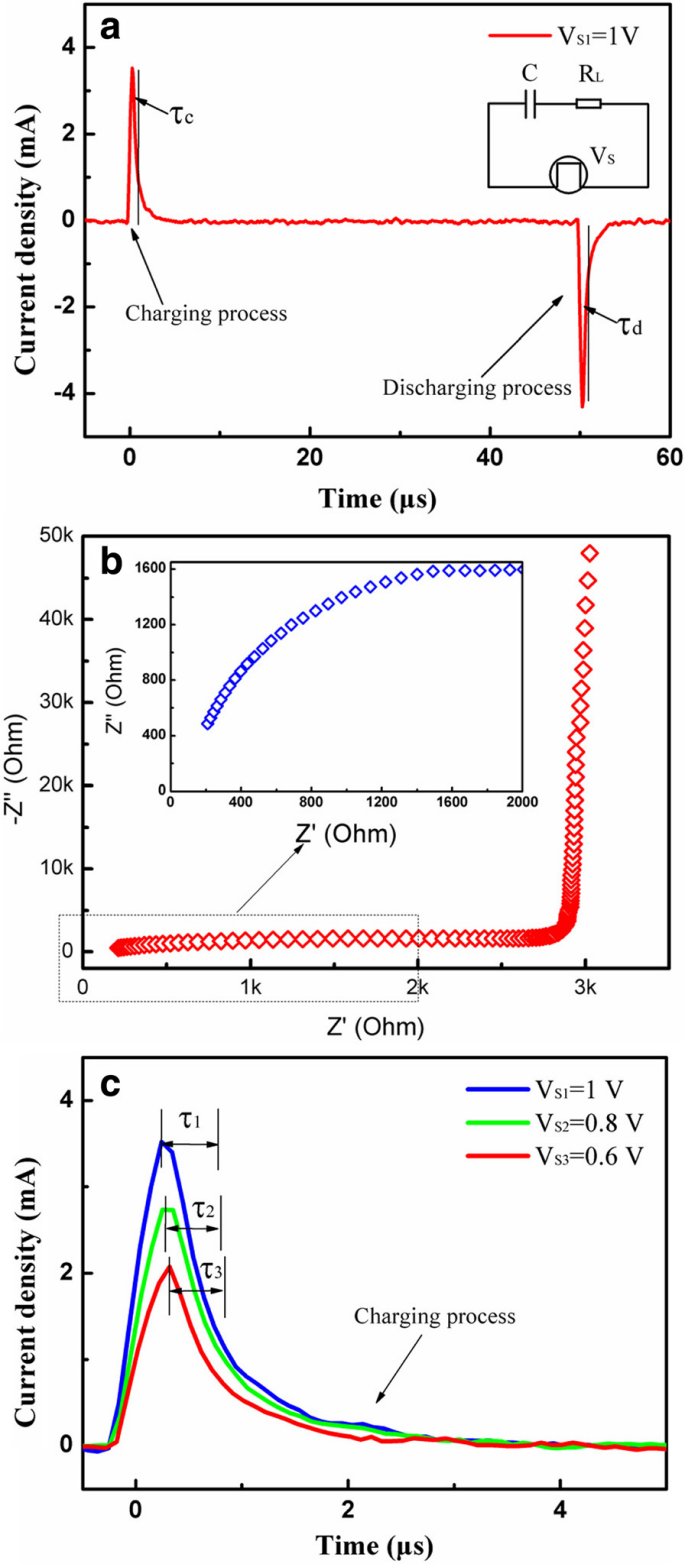
**a** Charge-discharge curve of the fabricated capacitor with 5 nm of Al_2_O_3_ together with the equivalent RC circuit. **b** Impedance spectrum of the capacitor, which was measured at a dc bias of 0 V with an AC signal of 5 mV over a frequency range from 1 MHz to 1 kHz. **c** The charging process of the corresponding fabricated capacitor with different voltages. The used pad area is 200 × 200 μm^2^

According to the charge and discharge processes of the resistor-capacitor circuits, the charge-discharge rate of the capacitor and hence the power characteristics of the capacitors are determined by the resistance-capacitance (RC) time constant [36]. The RC time constant (*τ*) is defined as the duration when the circuit current comes to e^−1^ (36.8 %) of the initial value. Figure 7a shows the charge-discharge curve of the fabricated capacitor with a 5-nm Al_2_O_3_ film and the equivalent RC circuit. The time constant is defined by *τ*_c_ and *τ*_d_ in the charging and discharging processes, respectively. When a 1 V bias is applied to the top electrode, both *τ*_c_ and *τ*_d_ are approximately equal to 550 ns; such short time constants are in accordance with the power characteristics of the electrostatic capacitor. The impedance analysis was carried out to estimate the internal resistance of the nanocapacitors, and the obtained impedance spectrum is shown in Fig. 7b. The curve shows a very steep behavior in the low frequency region, revealing good capacitor characteristics. The inset shows a magnified view of the impedance spectrum in the high frequency region, and the intercept at the real part axis indicates an equivalent series resistance (ESR) of the capacitor, revealing an ESR of about 110 Ω. Then, taking the load resistance (*R*_L_ = 50 Ω) and capacitance into consideration, the time constant was estimated to be 590 ns, which is close to the measurement result. Figure 7c shows the relationship between the charging current and time under different voltage supplies, where *V*_S1_ = 1 V, *V*_S2_ = 0.8 V, and *V*_S3_ = 0.6 V and the resulting time constants *τ*_1_, *τ*_2_, and *τ*_3_ are 550 ns. The results reveal that the time constant has nothing to do with the voltage supply, which is in agreement with the RC charge-discharge theory. The fabricated capacitors could obtain a high capacitance density, without sacrificing the power characteristics of the electrostatic capacitor.

## Conclusions

In summary, high-density 3D solid-state nanocapacitors based on stand-up ZnO NWs were fabricated successfully for the first time. For a 5-nm Al_2_O_3_ insulator, the capacitor had a high EPCD of up to 92 fF/μm^2^, an extremely low leakage current density of 3.4 × 10^−8^ A/cm^2^ at 2 V, and an RC time constant of 550 ns. These data reveal that the fabricated nanocapacitors have a high capacitance density, good power characteristics, and a low power consumption. In particular, such a low leakage current density means that the current nanocapacitor structure is very promising for energy storage applications. All the fabrication steps were carried out with a maximum processing temperature of 200 °C; thus, this can facilitate the manufacture of nanocapacitors on flexible substrates.
